# Associations between Urinary Phthalate Metabolites with BDNF and Behavioral Function among European Children from Five HBM4EU Aligned Studies

**DOI:** 10.3390/toxics12090642

**Published:** 2024-08-31

**Authors:** Elena Salamanca-Fernández, Lydia Espín-Moreno, Alicia Olivas-Martínez, Ainhoa Pérez-Cantero, José L. Martín-Rodríguez, Rafael M. Poyatos, Fabio Barbone, Valentina Rosolen, Marika Mariuz, Luca Ronfani, Ľubica Palkovičová Murínová, Lucia Fábelová, Tamás Szigeti, Réka Kakucs, Amrit K. Sakhi, Line S. Haug, Birgitte Lindeman, Janja Snoj Tratnik, Tina Kosjek, Griet Jacobs, Stefan Voorspoels, Helena Jurdáková, Renáta Górová, Ida Petrovičová, Branislav Kolena, Marta Esteban, Susana Pedraza-Díaz, Marike Kolossa-Gehring, Sylvie Remy, Eva Govarts, Greet Schoeters, Mariana F. Fernández, Vicente Mustieles

**Affiliations:** 1 Biomedical Research Center (CIBM), Department of Radiology and Physical Medicine, University of Granada, 18012 Granada, Spain; esalamanca@ugr.es (E.S.-F.); apercan@ugr.es (A.P.-C.); marieta@ugr.es (M.F.F.); 2 Instituto de Investigación Biosanitaria (ibs.GRANADA), 18012 Granada, Spain; aolivas@ugr.es; 3 CIBER de Epidemiología y Salud Pública (CIBERESP), 28034 Madrid, Spain; lydia.espin@ciberesp.es; 4 Servicio de Radiodiagnóstico, Hospital Universitario Clínico San Cecilio, 18012 Granada, Spain; 5 Unidad de Gestión Clínica de Laboratorios, Hospital Universitario Clínico San Cecilio, 18012 Granada, Spain; 6 Department of Medicine, Surgery and Health Sciences, University of Trieste, Strada di Fiume 447, 34149 Trieste, Italy; 7 Central Directorate for Health, Social Policies and Disability, Friuli Venezia Giulia Region, Via Cassa di Risparmio 10, 34121 Trieste, Italy; 8 Department of Environmental Medicine, Faculty of Public Health, Slovak Medical University, 831 04 Bratislava, Slovakia; 9 Center for Public Health and Pharmacy, Albert Flórián út 2-6, 1097 Budapest, Hungary; 10 Norwegian Institute of Public Health, P.O. Box 222, Skøyen, N-0213 Oslo, Norway; 11 Jožef Stefan Institute, 1000 Ljubljana, Slovenia; 12 VITO GOAL, Flemish Institute for Technological Research (VITO), Boeretang 200, 2400 Mol, Belgium; 13 Department of Analytical Chemistry, Faculty of Natural Sciences, Comenius University, Ilkovičova 6, Mlynská Dolina, 84215 Bratislava, Slovakia; 14 Department of Zoology and Anthropology, Faculty of Natural Sciences and Informatics, Constantine the Philosopher University in Nitra, Nabrezie mladeze 91, 94974 Nitra, Slovakia; 15 National Centre for Environmental Health, Instituto de Salud Carlos III, 28034 Madrid, Spain; 16 German Environment Agency (UBA), Corrensplatz 1, 14195 Berlin, Germany; 17 VITO Health, Flemish Institute for Technological Research (VITO), 2400 Mol, Belgium; 18 Department of Biomedical Sciences, University of Antwerp, 2610 Antwerp, Belgium

**Keywords:** phthalate, behavior, BDNF, effect biomarker, exposure biomarker, HBM4EU, PARC

## Abstract

Based on toxicological evidence, children’s exposure to phthalates may contribute to altered neurodevelopment and abnormal regulation of brain-derived neurotrophic factor (BDNF). We analyzed data from five aligned studies of the Human Biomonitoring for Europe (HBM4EU) project. Ten phthalate metabolites and protein BDNF levels were measured in the urine samples of 1148 children aged 6–12 years from Italy (NACII-IT cohort), Slovakia (PCB-SK cohort), Hungary (InAirQ-HU cohort) and Norway (NEBII-NO). Serum BDNF was also available in 124 Slovenian children (CRP-SLO cohort). Children’s total, externalizing and internalizing behavioral problems were assessed using the Child Behavior Checklist at 7 years of age (only available in the NACII-IT cohort). Adjusted linear and negative binomial regression models were fitted, together with weighted quantile sum (WQS) regression models to assess phthalate mixture associations. Results showed that, in boys but not girls of the NACII-IT cohort, each natural-log-unit increase in mono-n-butyl phthalate (MnBP) and Mono(2-ethyl-5-oxohexyl) phthalate (MEOHP) was cross-sectionally associated with higher externalizing problems [incidence rate ratio (IRR): 1.20; 95% CI: 1.02, 1.42 and 1.26; 95% CI: 1.03, 1.55, respectively]. A suggestive mixture association with externalizing problems was also observed per each tertile mixture increase in the whole population (WQS—IRR = 1.15; 95% CI: 0.97, 1.36) and boys (IRR = 1.20; 95% CI: 0.96, 1.49). In NACII-IT, PCB-SK, InAirQ-HU and NEBII-NO cohorts together, urinary phthalate metabolites were strongly associated with higher urinary BDNF levels, with WQS regression confirming a mixture association in the whole population (percent change (PC) = 25.9%; 95% CI: 17.6, 34.7), in girls (PC = 18.6%; 95% CI: 7.92, 30.5) and mainly among boys (PC = 36.0%; 95% CI: 24.3, 48.9). Among CRP-SLO boys, each natural-log-unit increase in ∑DINCH concentration was associated with lower serum BDNF levels (PC: −8.8%; 95% CI: −16.7, −0.3). In the NACII-IT cohort, each natural-log-unit increase in urinary BDNF levels predicted worse internalizing scores among all children (IRR: 1.15; 95% CI: 1.00, 1.32). Results suggest that (1) children’s exposure to di-n-butyl phthalate (DnBP) and di(2-ethylhexyl) phthalate (DEHP) metabolites is associated with more externalizing problems in boys, (2) higher exposure to DINCH may associate with lower systemic BDNF levels in boys, (3) higher phthalate exposure is associated with higher urinary BDNF concentrations (although caution is needed since the possibility of a “urine concentration bias” that could also explain these associations in noncausal terms was identified) and (4) higher urinary BDNF concentrations may predict internalizing problems. Given this is the first study to examine the relationship between phthalate metabolite exposure and BDNF biomarkers, future studies are needed to validate the observed associations.

## 1. Introduction

Phthalates are a family of industrial chemicals produced at high volumes and used as plasticizers in food packaging materials, medical equipment, toys, furniture and as solubilizers in cosmetics [1]. High-molecular-weight phthalates, such as di(2-ethylhexyl) phthalate (DEHP), are mainly used as plasticizers to impart flexibility in polyvinyl chloride (PVC) materials [2], while low-molecular-weight phthalates, such as di-ethyl phthalate (DEP), are often used as solvents in personal care products and in adhesives, varnishes and coatings [3]. In recent years, other phthalates including diisononyl phthalate (DiNP), and the non-phthalate plasticizer di-iso-nonyl-cyclohexane-1,2-dicarboxylate (DINCH^®^) are replacing DEHP and other phthalate compounds in several applications [4,5,6,7]. Human phthalate exposure occurs through multiple pathways [8], and although these compounds are rapidly metabolized and excreted in urine, exposure is chronic on a daily basis. Thus, more than 90% of the European population shows detectable urinary phthalate metabolite concentrations [9,10], and recent human biomonitoring studies also show increasing temporal trends for some phthalate replacements [9,11,12,13].

Some phthalates and their metabolites are known endocrine-disrupting chemicals (EDCs) [14,15,16,17], that is, chemicals or mixtures of chemicals able to interfere with different aspects of hormone action [18]. Additionally, some phthalates are known or suspected neurotoxicants [19]. Thus, exposure to both individual phthalate compounds and their mixtures has been shown to alter brain function and behavior in rodent models [20], often in a sex-specific manner [21]. In humans, exposure to specific phthalate metabolites has been associated with altered behavior, although more evidence is needed to confirm this relationship [22,23].

Phthalates and their metabolites can act through diverse modes of action, including anti-androgenic, estrogenic, thyroid-disrupting and/or oxidative stress pathways [24,25,26]. Using an adverse outcome pathway (AOP) network, we showed that the regulation of brain-derived neurotrophic factor (BDNF) is a downstream key central event of these mechanisms, having a key role in neurodevelopment [27]. BDNF is strongly involved in neuronal plasticity mechanisms, promoting neuronal growth, synaptic development and neuronal survival [28]. Peripheral BDNF biomarkers measured in plasma or serum have been shown to correlate with central BDNF levels in rodents [29]. Bisphenol A, certain heavy metals and nonpersistent pesticides have been associated with altered BDNF regulation in adolescent boys from the Spanish INMA-Granada cohort [28,30,31], and BDNF was shown to mediate the longitudinal association between BPA and altered behavior [28]. There is also preliminary evidence that altered BDNF regulation may also be an intermediate step between phthalate and neurodevelopmental impairments in rodents [32,33], although data are still lacking in epidemiological studies.

The European Human Biomonitoring Initiative [34] had among its objectives the assessment of exposure to priority environmental chemicals among Europeans in a harmonized manner. In practice, this was achieved in the so-called HBM4EU aligned studies [35,36,37], among other European populations. HBM4EU included phthalates as priority substances in order to further review their regulation in the EU [38]. In addition to exposure biomarkers, HBM4EU implemented effect biomarkers in relation to some of the health effects of concern. Indeed, BDNF was selected in relation to neurodevelopment [27,39,40]. This work is framed as a continuation of the research lines initiated in HBM4EU, now included within the objectives of the European project Partnership for the Assessment of Risks from Chemicals (PARC) [41]. The aim of this study was to investigate (1) whether children’s exposure to phthalates, both individually and as a mixture, is associated with alterations in their behavior, (2) if children’s exposure to phthalates is associated with BDNF levels and (3) whether BDNF levels can predict children’s behavioral problems.

## 2. Materials and Methods

### 2.1. Study Population

The present work used individual data concerning the assessment of child behavior, urinary and serum BDNF levels and urinary phthalate concentrations in five European cohorts/countries participating in the HBM4EU aligned studies. Studies included the Italian Northern Adriatic Cohort II (NACII-IT) study [42,43], the Slovak PCB-SK cohort [44], the Hungarian Integrated Indoor Air Quality Management (InAirQ-HU) cohort [45,46], the Norwegian Environmental Biobank Part II (NEBII-NO) cohort [47] and the Exposure of Children and Adolescents to Selected Chemicals Through Their Habitat Environment in Slovenia (CRP-SLO) cohort [48,49]. A detailed description of the sampling methods for the inclusion of participants and data harmonization in the HBM4EU aligned studies can be found elsewhere [35,36]. In brief, the study participants were 6–8-years-old children enrolled in the NACII-IT cohort, 8–11 years in the InAirQ-HU cohort, 10–12 years in the PCB-SK cohort, 7–11 years in the NEBII-NO cohort and 7–10 years in the CRP-SLO cohort. Information on the urinary concentrations of phthalate metabolites and urinary BDNF levels was available in four cohorts (NACII-IT, PCB-SK, InAirQ-HU and NEBII-NO), one cohort had data on urinary phthalate metabolites and serum BDNF levels (CRP-SLO) and NACII-IT also had data for child behavior. The selection of the participants of each cohort was performed following a step-wise selection procedure previously described [35]. Informed consent was obtained from the caregivers of all children involved, in accordance with the Declaration of Helsinki. The research protocols of the cohorts were approved by their respective ethics committees.

### 2.2. Exposure Biomarker Assessment

Concurrently with the behavioral evaluation, metabolites of the following parent phthalate compounds were measured in spot urine samples, previously collected in propylene tubes and stored between −20 and −80 °C: di-isobutyl phthalate (DiBP), di-n-butyl phthalate (DnBP), benzyl-butyl phthalate (BBzP), di-ethyl phthalate (DEP), di-(2-ethylhexyl) phthalate (DEHP) and di(isononyl)cyclohexane-1,2-dicarboxylate (DINCH)/HEXAMOLL^®^ metabolites. The following biomarkers of exposure were measured in the children’s urine: mono-isobutyl phthalate (MiBP) for DiBP, mono-n-butyl phthalate (MnBP) for DnBP, mono-benzyl phthalate (MBzP) for BBzP, mono-ethyl phthalate (MEP) for DEP, mono(2-ethyl-5-hydroxyhexyl) phthalate (MEHHP), mono(2-ethyl-5-oxohexyl) phthalate (MEOHP) and mono(2-ethyl-5-carboxypentyl) phthalate (MECPP) for DEHP. Mono(2-ethylhexyl) phthalate (MEHP) was not measured in the NACII-IT cohort, and therefore in this work, we focused on the other three DEHP metabolites (MEHHP, MEOHP and MECPP). Cyclohexane-1,2-dicarboxylic acid monohydroxy isononyl ester (MHINCH) and cyclohexane-1,2-dicarboxylic acid monocarboxyisooctyl ester (MCOCH) metabolites were measured to cover exposure to the non-phthalate replacement DINCH (HEXAMOLL^®^). The NEBII-NO cohort had data on MHINCH but not on MCOCH.

The quality and inter-comparability of results from the laboratories that measured the urinary phthalate and DINCH metabolites were assured through the HBM4EU quality control (QA/QC) program [50,51]. The participating laboratories in the HBM4EU analyzed standard test materials using their own method, in all cases using liquid chromatography-tandem mass spectrometry (LC-MS/MS). Laboratories reported the concentrations in ng/mL through a web tool and provided the details of their methods (e.g., regarding deconjugation, extraction, instrumental analysis, use of internal standards, method of quantification and identification parameters). Extended information of the phthalates and DINCH exercises can be found elsewhere [51]. All samples were analyzed in the laboratories that participated and obtained successful results in the QA/QC HBM4EU program [50,51]. Details for each specific cohort have been previously described [52]. In order to improve comparability, urinary creatinine was measured to standardize phthalate metabolites by the voiding volume (urine dilution) of the participants.

### 2.3. Effect Biomarker Assessment: Urinary and Serum BDNF Levels

Urinary BDNF protein levels were measured in four out of the five included cohorts: NACII-IT, PCB-SK, InAirQ-HU and NEBII-NO, at the Hospital Universitario Clinico-San Cecilio (HUSC) of Granada (Spain), using the commercial RayBio^®^ ELISA kit (Raybiotech, Norcross, GA, USA). This ELISA kit was the one that proved to offer the best performance among the several kits tested within a previous HBM4EU validation program for effect biomarkers [53]. Briefly, urine samples were thawed, vortexed and pretreated following the protocol and procedure described in Olivas-Martínez et al. [53]. Each urine sample was assessed in duplicate in different batches, and the mean value was calculated. The intra- and inter-assay coefficients of variability were <5% and <15%, respectively. 

Serum total BDNF protein levels were measured in the CRP-SLO cohort using the commercial Quantikine^®^ enzyme-linked immunosorbent assay (ELISA) kit (R&D Systems, Minneapolis, MN, USA). Serum samples were defrosted, vortexed, separated in two aliquots of 10 μL, diluted 100 times and tested according to the manufacturer’s instructions at the Biomedical Research Center (CIBM) of the University of Granada (Spain). Each sample was tested in duplicate in different plates, and the mean of these two values was calculated in order to reduce measurement variation. The intra- and inter-assay coefficients of variability were <5% and <15%, respectively.

### 2.4. Behavioral Assessment in the NACII-IT Cohort

In the NACII-IT cohort, and concurrently with the collection of urine samples, children’s behavioral function was assessed using the parent-reported Child Behavior Checklist (CBCL/6-18), a validated questionnaire that evaluates parental perception about the behavior of their children and/or adolescents during the previous six months [54]. The CBCL includes 118 items rated on a three-point Likert scale (0 = “Not True”, 1 = “Somewhat or Sometimes True” or 2 = “Very/Often True”) which are grouped into eight syndrome scales (anxious/depressed, withdrawn/depressed, somatic complaints, social problems, thought problems, attention problems, rule-breaking behavior and aggressive behavior). These scales are also grouped into two empirically derived composite scales: (i) the internalizing domain as a measure of emotional problems (sum of scores on the anxious/depressed, withdrawn/depressed and somatic complaints scales) and (ii) the externalizing domain as a measure of behavioral problems (sum of scores on the rule-breaking behavior and aggressive behavior scales). Three other scales are considered mixed-syndrome scales that do not belong to either domain: social, thought and attention problems. The total problems composite scale finally quantifies general impairment and corresponds to the sum of scores from the eight syndrome scales, together with a group of 17 “Other problems” items that do not belong to any specific syndrome scale. Our analysis was focused on continuous raw scores for the total [range 0–87], externalizing [range 0–27] and internalizing [range 0–34] composite scales, with higher scores meaning higher behavioral problems.

### 2.5. Covariates

As previously described [55], based on information obtained from the questionnaires applied in the different HBM4EU aligned studies [35,36], covariates were harmonized. The level of education of the mothers was based on the International Standard Classification of Education (ISCED) developed by the United Nations Educational, Scientific and Cultural Organization (UNESCO) [56]. Mothers with less than secondary education were included in the lower-education level (ISCED 0–2), mothers with secondary but not postsecondary education represented the medium level (ISCED 3–4) and those with college and/or university degrees and higher education were included in the high-education level (ISCED ≥ 5). At urine sample collection, the children’s height and weight were measured by the healthcare staff in each cohort (with the exception of the NEBII-NO cohort in which self-reported measures were provided). BMI (kg/m^2^) was calculated as the ratio between weight (kg) and height squared (m^2^). In order to improve comparability among studies, age- and sex-specific BMI z-scores were calculated using the 2007 World Health Organization (WHO) growth reference standards [57]. Child sex, age and season of urine collection were obtained from questionnaires. Maternal intelligence quotient (IQ) was assessed using Raven’s Progressive Matrices Test and was considered in models examining CBCL scores, only available in the NACII-IT cohort.

### 2.6. Statistical Analysis

Study participant characteristics were described using measures of central tendency and dispersion for numerical variables and frequencies for categorical variables.

#### 2.6.1. Phthalate Exposure Biomarkers

All selected phthalate and DINCH metabolites were detected in most urine samples (>93%). The few concentrations below the limit of detection (LOD) were singly and randomly imputed considering the distribution of quantified values [58]. For phthalate metabolites belonging to a same parent compound, molar sums (µmol/L) were also computed (i.e., ∑DEHP and ∑DINCH). The ∑DINCH measure was estimated without considering the NEBII-NO cohort which only had data on the MHINCH but not the MCOCH metabolite. Urinary phthalate metabolite concentrations were divided or standardized by urinary creatinine levels and expressed as micrograms of chemical per gram of creatinine. Creatinine-standardized urinary phthalate metabolites were natural log transformed to reduce the skewness of distributions and the influence of extreme values. Correlation among phthalate metabolites was examined using Spearman’s rank correlation coefficients.

#### 2.6.2. Urinary and Serum BDNF Protein Levels

Urinary BDNF protein levels were standardized by urinary creatinine levels and expressed as micrograms of BDNF per gram of creatinine. Both serum BDNF protein levels and creatinine-standardized urinary BDNF levels were natural log transformed to reduce the skewness of distributions and the influence of extreme values.

#### 2.6.3. Covariates and Confounding Factors

Potential confounders of phthalate–BDNF behavior associations were identified a priori based on causal scientific knowledge [59]. All models were adjusted for the same set of covariates: maternal education [low (0–2), middle (3–4) or high (>=5) ISCED classification], child sex, age at urine collection or CBCL assessment, child body mass index z-score (BMI z-score), season of urine collection (summer/autumn vs. winter/spring, since specific CBCL scores and levels of some phthalates differed according to season) and creatinine (log transformed). The phthalate–CBCL and BDNF–CBCL models were additionally adjusted for maternal IQ (but only in the NACII-IT cohort, which had data on CBCL behavior scores concurrently with phthalate and BDNF measurements). Phthalate associations with urinary BDNF levels were examined in four cohorts (NACII-IT, PCB-SK, InAirQ-HU and NEBII-NO), including “cohort” as an additional covariate in the statistical models. The slight number of missing values for some covariates (in all cases <4%) was singly imputed with the variable median or mode value.

#### 2.6.4. Cross-Sectional Associations between Phthalates, BDNF Concentrations and Children’s Behavior

As a first step, we only analyzed data from the NACII-IT cohort, since it had data on phthalate metabolites, urinary BDNF concentrations and CBCL scores: (1) phthalate–CBCL associations were examined using adjusted negative binomial regression (NBR) [60,61,62]. Since phthalate concentrations were natural log transformed prior to analysis, the incidence rate ratios (IRRs) and 95% confidence intervals (95% CIs) corresponded to the multiplicative change in the probability of CBCL scores for each natural log-unit increase in exposure biomarkers; (2) the phthalate–BDNF associations were examined using adjusted linear regression models. Since both phthalate metabolites and BDNF were natural log transformed, regression estimates were expressed as the percent change (PC) for each natural-log-unit increase in standardized urinary phthalate biomarkers using the following formula: ([e^(β)^ − 1] × 100); (3) BDNF–CBCL associations were also examined using NBR models.

As a second step, the phthalate–BDNF associations were investigated together in the four cohorts that had data on urinary BDNF levels, using linear regression models and expressing results as the PC for each natural-log-unit increase in standardized urinary phthalate biomarkers.

A phthalate–sex interaction term was added to the models to identify potential sex-specific associations, since previous studies have shown effect modification by this variable [21,23]. Nevertheless, considering the modest sample size, especially for behavior analyses only available in the NACII-IT cohort, an interaction term *p*-value below 0.10 was considered as potential evidence of effect modification.

#### 2.6.5. Phthalate’s Mixture Models

The overall association between childhood exposure to mixtures of phthalates and DINCH metabolites on urinary BDNF levels and behavioral CBCL scores was estimated using adjusted linear and negative binomial weighted quantile sum (WQS) regressions for BDNF and CBCL scores, respectively. WQS regression was selected since it allowed to model mixture effects using negative binomial regression. Consistent with single-pollutant models, WQS regressions were adjusted for the same set of covariates, and we fitted models for the whole population, as well as in boys and girls separately. Phthalate and DINCH metabolite concentrations were modeled in tertiles, and thus the results represent the change in estimates per tertile increase in the phthalate mixture.

WQS is a quantile-based approach that combines multiple independent variables additively into a weighted index, with weights estimating the contribution of each component to the mixture. WQS regression assumes that all exposures included in the index are associated with the outcome in the same direction [63,64]. Therefore, after observing no mixture effects in the negative direction, only WQS indexes in the positive direction were considered, in line with our initial hypothesis expecting deleterious effects of phthalate exposure on child behavior. WQS performs a random partition of the data into training and validation datasets, used to estimate the relative weights of each chemical/metabolite and test the statistical significance of the WQS index, respectively. Such splitting reduces statistical power and may lead to unstable results. To counteract this issue, we implemented a repeated holdout validation [65], consisting in repeating “n” times the random partitioning and WQS analyses and averaging the “n” estimates. We repeated WQS regressions 100 times; the study population was split into training (40%) and validation (60%) sets; and weights were calculated based on 100 bootstrap samples. The scatterplots of the WQS index vs. the outcome (adjusted for covariate model residuals) were checked to examine the shape of the mixture associations, which in this analysis did not show relevant departures from linearity.

#### 2.6.6. Sensitivity Analysis

Phthalate associations with urinary BDNF levels were studied separately in each of the four HBM4EU aligned studies to assess robustness.

Since different methods to account for urinary dilution could provide different results, we tested and compared five different approaches to adjust for urinary creatinine levels in models of urinary phthalates and urinary BDNF levels in the four HBM4EU aligned studies combined. The approaches tested include (a) raw phthalates and BDNF levels controlling for creatinine as a covariate, (b) phthalates and BDNF levels standardized by creatinine, (c) phthalate and BDNF levels standardized by creatinine in addition to adjusting for creatinine as a covariate (main model), (d) covariate-adjusted standardization (CAS) of both phthalates and BDNF levels, following the method described by O’Brien et al. [66], and (e) CAS standardization controlling for creatinine as a covariate.

#### 2.6.7. Interpretation and Software

Statistical significance was set as *p*-value < 0.05. A *p*-value between 0.05 and 0.10 was considered as possibly suggestive of an association. However, results were interpreted not solely depending on statistical significance but also considering patterns of associations and the previous evidence available to contextualize the observed findings [67]. Regression analyses were carried out with Stata version 14.2 (Stata Corp., College Station, TX, USA), while WQS models were implemented using the “gWQS” package (https://cran.r-project.org/web/packages/gWQS/gWQS.pdf, accessed on 28 August 2024) in RStudio version 4.0.3 (RStudio Team, 2020).

## 3. Results

### 3.1. Study Population Characteristics

A total of 1148 children were included in the current analysis (Table 1). Half (50%) of the children were boys, and the mean age (standard deviation—SD) and BMI z-score at the time of urine collection was 9.23 (1.7) years and 0.51 (1.17) kg/m^2^, respectively. Almost half (45%) of the mothers had a high-education level, with PCB-SK mothers having the lowest percentage (9% highly educated). The mean (SD) total CBCL raw score in NACII-IT was 23 (15), ranging from 0 to 87 points. In the four cohorts combined, urinary BDNF median concentrations (percentiles P25, P75) were 2.31 (1.52, 3.46) μg/g creatinine. In the SLO-CRP cohort, median (P25, P75) serum BDNF levels were 45.1 (38.9, 52.6) ng/mL. The highest median (P25, P75; μg/g creatinine) concentrations of the investigated phthalate and DINCH metabolites were observed for the sum of ∑DEHP metabolites [56.4 (35.6, 93.4)], followed by MiBP [33.9 (21.5, 57.7)], MnBP [26.3 (15.5, 47.2)] and MEP [21.8 (10.4, 54.4)], while MBzP [4.14 (1.80, 7.99)] and ∑DINCH [3.30 (1.68, 6.68)] showed the lowest concentrations (Table 1). Phthalate metabolite concentrations, including MEP, MnBP, ∑DEHP and ∑DINCH, differed across the cohorts, highlighting substantial variability linked to different countries and lifestyles (Table 1). The Spearman’s correlation coefficients among phthalate metabolites were in general low to moderate, with the exception of metabolites belonging to the same parent compound, which, as expected, showed strong correlations among them (Supplementary Figure S1).

### 3.2. Phthalate–BDNF–Behavior Associations in the NACII-IT Cohort

**Figure 1:**
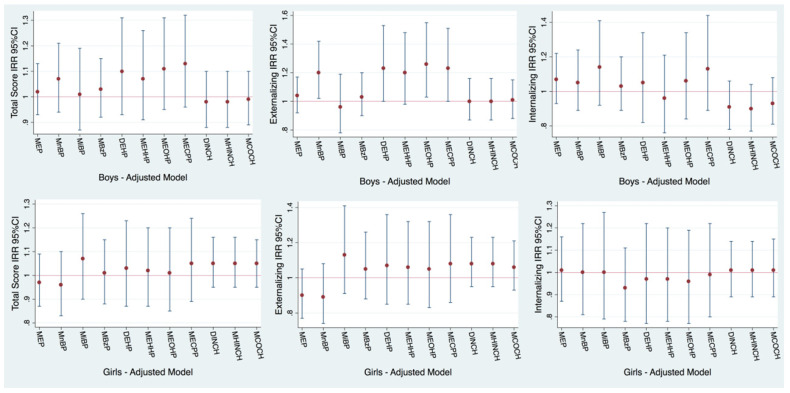
Adjusted cross-sectional associations between concurrent childhood urinary phthalate metabolite concentrations and CBCL scores in boys and girls from the NACII-IT cohort (n = 298). Note: Phthalate concentrations were natural log transformed, so the incidence rate ratio (IRR) and 95% confidence interval (95% CI) correspond to the multiplicative change in the probability of CBCL scores for each 2.7-fold increase in exposure biomarkers. Models adjusted for maternal education (low (0–2), middle (3–4) or high (>=5) ISCED classification), child body mass index (BMI) z-score, creatinine (log transformed), child age at CBCL assessment, season of urine collection (summer/autumn vs. winter/spring) and maternal IQ. The numeric results corresponding to this figure are available in Supplementary Table S2.

No associations were observed between phthalate metabolites and CBCL scores when boys and girls were analyzed together (Supplementary Table S1). However, among boys, each natural-log-unit increase in urinary MnBP and DEHP metabolite concentrations was associated with higher externalizing behavior scores (Figure 1, Supplementary Table S2): MnBP (IRR: 1.20; 95% CI: 1.02, 1.42), ∑DEHP (IRR: 1.23; 95% CI: 1.00, 1.53), MEHHP (IRR: 1.20; 95% CI: 0.98, 1.48), MEOHP (IRR: 1.26; 95% CI: 1.03, 1.55) and MECPP (IRR: 1.23; 95% CI: 1.00, 1.51). Regarding the MnBP–externalizing behavior association, evidence of effect modification by sex was found (*p*-interaction: 0.01), with boys showing higher externalizing scores (IRR: 1.20; 95% CI: 1.02, 1.42) as abovementioned, and girls showing a nonsignificant opposite trend toward lower externalizing scores (IRR: 0.89; 95% CI: 0.74, 1.08) (Figure 1, Supplementary Table S2).

**Figure 2:**
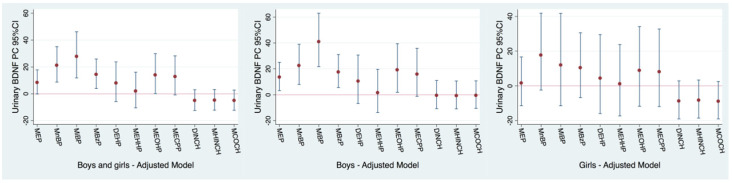
Adjusted cross-sectional associations between concurrent childhood urinary phthalate metabolite concentrations and urinary BDNF levels in children from the NACII-IT cohort (n = 299). Note: Phthalate concentrations were natural log transformed, so the percent change (PC) and 95% confidence interval (95% CI) correspond to the mean change in the urinary BDNF levels for each 2.7-fold increase in exposure biomarkers. Models adjusted for maternal education (low (0–2), middle (3–4) or high (>=5) ISCED classification), child body mass index (BMI) z-score, creatinine (log transformed), child sex, child age at urine collection and season of urine collection (summer/autumn vs. winter/spring). The numeric results corresponding to this figure are available in Supplementary Table S3.

Each natural-log-unit increase in several urinary phthalate metabolites was associated with higher urinary BDNF levels among both boys and girls from the NACII-IT cohort (Figure 2 and Supplementary Table S3): MnBP (PC: 21.2%; 95% CI: 8.74, 35.1), MiBP (PC: 27.8%; 95% CI: 11.8, 46.2), MBzP (PC: 14.4%; 95% CI: 3.93, 25.9) and MEOHP (PC: 14.0%; 95% CI: 0.11, 29.9). When sex-stratified, boys showed stronger and significant associations with higher urinary BDNF levels for MEP, MnBP, MiBP, MBzP and MEOHP, while only MnBP was borderline-associated with higher BDNF levels among girls (Figure 2, Supplementary Table S3).

**Figure 3:**
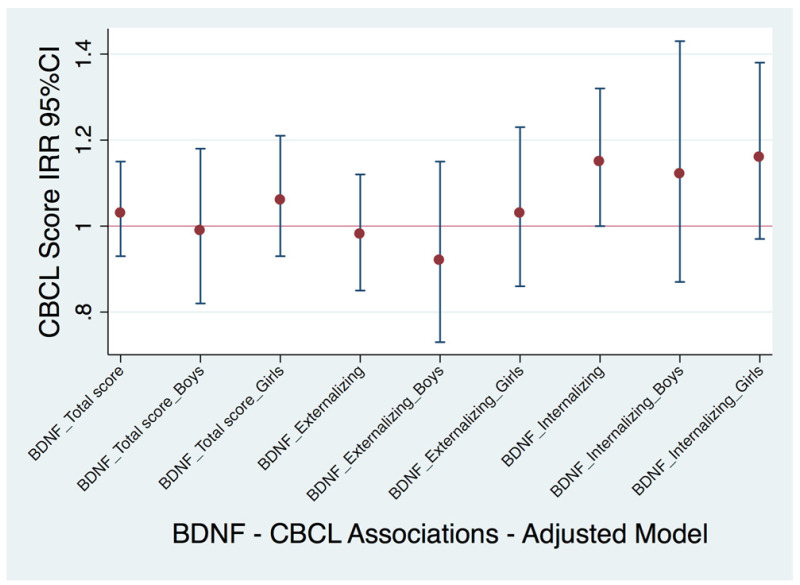
Adjusted cross-sectional associations between concurrent childhood urinary BDNF levels and CBCL scores in children from the NACII-IT cohort (n = 299). Note: BDNF levels were natural log transformed, so the incidence rate ratio (IRR) and 95% confidence interval (95% CI) correspond to the multiplicative change in the probability of CBCL scores for each 2.7-fold increase in urinary BDNF levels. Models adjusted for maternal education (low (0–2), middle (3–4) or high (>=5) ISCED classification), child body mass index (BMI) z-score, creatinine (log transformed), child age at CBCL assessment, season of urine collection (summer/autumn vs. winter/spring) and maternal IQ. The numeric results corresponding to this figure are available in Supplementary Table S4.

When boys and girls from the NACII-IT cohort were considered together, each natural-log-unit increase in urinary BDNF levels was associated with higher internalizing problems (IRR: 1.15; 95% CI: 1.00, 1.32) but not with externalizing or total CBCL scores (Figure 3, Supplementary Table S4).

### 3.3. Phthalate–Urinary BDNF Associations in NACII-IT, PCB-SK, InAirQ-HU and NEBII-NO Cohorts (N = 1148)

**Figure 4:**
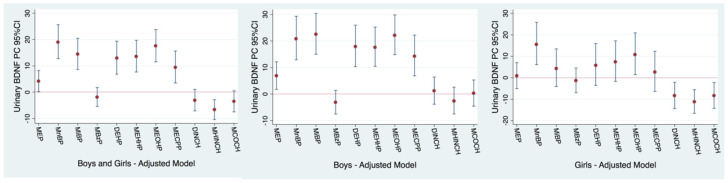
Adjusted cross-sectional associations between concurrent childhood urinary phthalate metabolite concentrations and urinary BDNF levels in all children from the NACII-IT, PCB-SK, InAirQ-HU and NEBII-NO cohorts (N = 1148). Note: Phthalate concentrations were natural log transformed, so the percent change (PC) and 95% confidence interval (95% CI) correspond to the mean change in the urinary BDNF levels for each 2.7-fold increase in exposure biomarkers. Models adjusted for maternal education (low (0–2), middle (3–4) or high (>=5) ISCED classification), child body mass index (BMI) z-score, creatinine (log transformed), child sex, child age at urine collection and season of urine collection (summer/autumn vs. winter/spring). The ∑DINCH measure was estimated without considering the NEBII-NO cohort, which only had data on the MHINCH but not the MCOCH metabolite. The numeric results corresponding to this figure are available in Supplementary Table S4.

When the four cohorts with available urinary BDNF measures were analyzed together, with the exception of DINCH metabolites, each natural-log-unit increase in most urinary phthalate metabolites was strongly associated with higher urinary BDNF levels among both boys and girls together (Figure 4 and Supplementary Table S5): MEP (PC: 4.2%; 95% CI: 0.20, 8.3), MnBP (PC: 19.0%; 95% CI: 12.7, 25.6), MiBP (PC: 14.4%; 95% CI: 8.6, 20.5), ∑DEHP (PC: 13.0%; 95% CI: 6.8, 19.4), MEHHP (PC: 13.5%; 95% CI: 7.7, 19.7), MEOHP (PC: 17.5%; 95% CI: 11.6, 23.8) and MECPP (PC: 9.4%; 95% CI: 3.5, 15.7). Sex-stratified models showed that, among boys, all the previous associations were strengthened (Figure 4 and Supplementary Table S5), while in girls, only MnBP and MEOHP were positively associated. Among girls, inverse associations were also observed between higher urinary DINCH metabolites and lower urinary BDNF levels (Figure 4 and Supplementary Table S5): DINCH (PC: −8.4%; 95% CI: −14.3, −2.2), MHINCH (PC: −11.3%; 95% CI: −16.6, −5.6) and MCOCH (PC: −8.4%; 95% CI: −14.3, −2.2).

### 3.4. Phthalate–Serum BDNF Associations in the CRP-SLO Cohort (N = 124)

In boys but not girls of the CRP-SLO cohort, higher urinary concentrations of ∑DINCH and their metabolites were associated with lower serum BDNF levels: ∑DINCH (PC: −8.8%; 95% CI: −16.7, −0.3), MHINCH (PC: −7.9; 95% CI: −15.6, 0.4) and MCOCH (PC: −10.1%; 95% CI: −18.0, −1.5) (Supplementary Table S6). In girls, a borderline-significant trend toward lower serum BDNF levels (PC: −6.83%; 95% CI: −14.0, 0.98) was also noted in relation to higher urinary MiBP concentrations (Supplementary Table S6).

### 3.5. Phthalate’s Mixture Associations

Regarding CBCL scores in the NACII-IT cohort, WQS regression models showed that each tertile increase in the phthalate mixture was suggestively associated with higher externalizing scores in the whole population (IRR = 1.15; 95% CI: 0.97, 1.36), mostly driven by boys (IRR = 1.20; 95% CI: 0.96, 1.49) (Table 2). Both in the overall population and among boys, MEOHP, MnBP and MCOCH metabolites were identified as the main contributors to the mixture effect (Supplementary Figure S2).

Regarding urinary BDNF levels in the four cohorts together, WQS regression models showed that each tertile increase in the phthalate mixture was significantly associated with higher BDNF levels in the whole population (PC = 25.9%; 95% CI: 17.6, 34.7), also in girls (PC = 18.6%; 95% CI: 7.92, 30.5) and especially among boys (PC = 36.0%; 95% CI: 24.3, 48.9). The main contributors to the mixture effect on urinary BDNF levels were the metabolites MEOHP, MEP, MnBP and MiBP (Supplementary Figure S2).

### 3.6. Sensitivity Analysis

When phthalate–BDNF associations were stratified by cohort, we observed higher urinary BDNF levels in response to higher phthalate metabolites in the NACII-IT cohort, followed by boys of the PCB-SK cohort (Figure 2). Mostly null associations were observed for the NEBII-NO and InAirQ-HU cohorts (Supplementary Figure S3).

Very similar results were observed for the five approaches used to account for urinary creatinine levels (Supplementary Figure S4), indicating that the positive associations observed between urinary phthalates and BDNF concentrations in children from the four European cohorts combined were robust to all types of creatinine adjustments. Indeed, the only associations that varied were the negative associations between DINCH metabolites and urinary BDNF levels, which were only observed in models that accounted for creatinine as a covariate.

## 4. Discussion

This cross-sectional study in European children including five HBM4EU aligned studies showed that (1) in the NACII-IT cohort, higher urinary concentrations of MnBP and DEHP metabolites were associated with externalizing problems among boys but not girls, while the phthalate mixture was suggestively associated with higher externalizing scores in both the whole population and boys; (2) most urinary phthalate metabolites, individually or as a mixture, were associated with higher urinary BDNF protein levels in NACII-IT, PCB-SK, InAirQ-HU and NEB-NO cohorts combined, with stronger associations found in boys; (3) higher urinary DINCH concentrations were associated with lower serum BDNF levels in boys of the CRP-SLO cohort and (4) higher urinary BDNF levels were associated with more internalizing—but not externalizing—problems among both boys and girls of the NACII-IT cohort.

### 4.1. Phthalate–CBCL Associations

Our observation that urinary concentrations of MnBP and DEHP metabolites, individually and as a mixture, were associated with higher externalizing problems in boys is consistent with previous epidemiological studies. For example, higher prenatal exposure to DEHP metabolites was associated with more externalizing problems among boys in one epidemiological study [68], and in both boys and girls in others [69,70]. Studies have also observed that higher maternal urinary DEHP concentrations during pregnancy were associated with more attention-deficit hyperactivity disorder (ADHD) symptoms among boys [71]. Breastmilk DEHP exposure was also associated with higher infant inattention and hyperactivity symptoms [72]. Higher prenatal exposure to MnBP was also associated with more externalizing [73], as well as with social and emotional problems among boys [74] and total behavioral problems in boys [75]. Additionally, the few postnatal studies performed to date have shown that concurrent exposure to DnBP metabolites was associated with total, emotional and hyperactivity behavioral problems in 7-year-old boys and girls [76] and that MnBP and DEHP metabolites were cross-sectionally associated with more social problems in boys [77]. In children, DEHP exposure has also been cross-sectionally associated with a higher risk of ADHD [78]. Notwithstanding, other studies reported null or contradictory associations [79,80]. Despite heterogeneity in exposure timing, sampling protocols and the different neurobehavioral tests and domains assessed, the epidemiological literature generally supports that developmental exposure to MnBP and DEHP metabolites is linked to poorer neurobehavioral scores among children.

Toxicological studies in rodents have also revealed effects of perinatal DEHP exposure on exploratory, social and anxiety behaviors [81,82,83,84,85,86,87]. Toxicological data for the effect of MnBP and its parent compound on the brain and behavior are scarce but also point in a similar direction [88].

Regarding possible modes of action, both MnBP and DEHP have shown anti-androgenic activities in both toxicological [87,88,89,90,91] and epidemiological studies [92,93], providing a potential clue as to why associations were observed in boys but not girls. Notwithstanding, it should be noted that these phthalates can also act through other modes of action including thyroid disruption, oxidative stress, peroxisome proliferator-activated receptors (PPARs) and estrogen pathways [25,94]. Overall, scientific findings including ours support that MnBP and DEHP metabolites, individually and as a mixture, may have a deleterious impact on children’s neurodevelopment, calling for further policies aiming at reducing children’s exposure to phthalates [19,95].

### 4.2. Urinary Phthalates and Serum BDNF Levels

The inverse associations found between exposure to DINCH metabolites and serum BDNF levels among boys of the CRP-SLO cohort suggest that exposure to this non-phthalate replacement may be related to lower systemic levels of BDNF. Since lower serum BDNF levels have been linked to a variety of neuropsychiatric disorders including depression and anxiety [96,97], further investigation of this phthalate substitute is warranted. Given the limited sample size and that this is the first human study investigating the DINCH–BDNF association, additional studies are needed to validate these results.

### 4.3. Urinary Phthalate–Urinary BDNF Associations—Possible “Urine Concentration Bias”?

This is the first epidemiological study that investigated phthalate exposure in relation to the neurological effect biomarker BDNF. Our results indicate consistently higher BDNF protein levels in urine in response to most phthalate metabolites. This association was additionally observed in phthalate mixture models, especially driven by boys. Since the analysis in the NACII-IT cohort also showed that higher urinary BDNF levels were predictive of more internalizing problems among boys and girls analyzed together, urinary BDNF concentrations may be linked to poorer neurobehavior. However, it is not clear whether higher urinary BDNF concentrations reflect higher or lower systemic BDNF levels, and even more complex, both low and high serum BDNF levels have been linked to altered neurobehavior, with lower levels associated with anxiety and depression [96,97], while high levels are linked to ADHD and autism spectrum disorders [98,99]. Caution is needed when interpreting associations with urinary BDNF levels because of the fact that most—although not all—phthalate metabolites showed strong and consistent associations toward higher urinary BDNF levels (a result not frequently observed in the environmental epidemiology field), that could also reflect a potential and unintentional noncausal artifact.

An alternative noncausal explanation could be that urine samples with a higher concentration (due to longer storage in the bladder or lower hydration status) in general would tend to correlate with higher phthalate metabolites and with higher physiological urinary waste products, including urinary BDNF levels. To test this hypothesis, we performed a post hoc analysis checking Spearman’s correlations between urinary creatinine in relation to phthalate/DINCH metabolites and BDNF levels (Supplementary Table S7). Results showed that urinary creatinine levels were correlated positively and significantly (*p* < 0.001) with urinary BDNF and all urinary phthalate/DINCH metabolites examined, with rho values ranging from 0.26 to 0.59 (Supplementary Table S6). This post hoc analysis supports the hypothesis that, in spot urine samples, urinary concentration tends to be correlated with higher exposure (phthalate/DINCH metabolites) and effect biomarker (BDNF) levels. This led us to formulate the following question: in studies measuring exposure and effect biomarkers in the same spot urine sample, could urine concentration favor the appearance of noncausal associations toward a positive direction, even when standardizing and adjusting models for creatinine such as in the current work?

Until now, most previous studies in the field have assessed exposure to phthalates, phenols and metals in relation to oxidative stress and kidney damage biomarkers measured in urine, in most cases finding positive associations. Examples include the review by Steffensen et al. reporting that urinary bisphenol A exposure was associated with higher urinary oxidative stress biomarkers in most of the studies evaluated [100]. Urinary metals have also been consistently linked to higher urinary oxidative stress and renal biomarkers [101,102,103], even showing contradictory associations between antioxidant essential metals such as selenium and higher urinary oxidative stress biomarkers [103]. Urinary phthalate metabolites have also been associated with higher oxidative stress in many studies [104,105,106,107]. Noteworthy, Davalos et al. were able to compare associations between urinary phthalates and oxidative stress biomarkers in urine samples collected at the same time or some days after exposure was assessed. In the cross-sectional evaluation, they found that many phthalate metabolites were associated with increased oxidative biomarkers. In the short-term longitudinal analysis, they still found some associations toward increased oxidative stress, but the magnitude of associations was importantly attenuated: for example, percent increase per interquartile range (IQR) difference in individual phthalate metabolites and the 8-iso-PGF2α metabolite ranged from 16% to 63% in cross-sectional models but only 2.9% to 9.8% in longitudinal models [104]. Therefore, these results suggest that although phthalate exposure may increase oxidative stress as shown by experimental laboratory models [108,109], human studies assessing phthalate metabolites and oxidative stress biomarkers in the same urine sample may considerably overestimate the magnitude of associations or even produce spurious positive associations. Against this alternative noncausal hypothesis is the fact that, in the current study, positive associations were not systematically observed in all cohorts, and even a few negative associations were observed in one of the studies examined (InAirQ-HU, Supplementary Figure S3), although this result was an exception compared to findings in the remaining cohorts.

Overall, our study raises a concern when exposure and effect biomarkers are measured in the same urine sample because urine concentration may tend to exert an effect that statistical methods are not able to disentangle, as shown in our dataset by a comparison of five different methods to account for urinary creatinine levels (Supplementary Figure S4). While additional methodological studies are needed to achieve a final response to this question, it is advisable that future biomarker studies, when possible, measure urinary exposure and effect biomarkers in urine samples separated in time [104] or measure the effect biomarkers of interest in blood or alternative matrices to urine in order to avoid this potential “urine concentration bias”.

### 4.4. BDNF Biomarkers in Relation to CBCL

Regardless of whether the observed urinary phthalate–urinary BDNF associations are causal or not, higher urinary BDNF levels appeared as a predictor of internalizing problems in both boys and girls of the NACII-IT cohort. This is relevant since current evidence supports that (a) BDNF can be reliably measured in urine [53], and (b) it has a physiological or pathophysiological meaning worthy of further examination in future epidemiological studies. Of note, we initially aimed to assess serum protein BDNF levels and blood BDNF DNA methylation in addition to urinary levels, but it was not possible due to the lack of availability of these biological matrices in the HBM4EU aligned studies in children (with the exception of serum BDNF levels in the CRP-SLO cohort). Measuring BDNF in urine has the advantage of being a noninvasive matrix widely available in human biomonitoring studies. Notwithstanding, in a previous HBM4EU pilot study using the INMA (Environment and Childhood) cohort from Granada (Southern Spain), we showed that blood BDNF DNA methylation was altered in response to bisphenol A, heavy metals and nonpersistent pesticide exposure [28,30,31], being apparently more useful compared to serum or urine BDNF protein levels. In the case of bisphenol A, BDNF DNA methylation levels even mediated about a third of the association observed between urinary bisphenol A concentrations measured at 9 years and behavior assessed using the CBCL test at 15 years of age [28]. More evidence is needed to identify which BDNF biomarkers show the best predictive potential for neurobehavioral outcomes; meanwhile, a combination of blood- and urine-based BDNF biomarkers may be advisable as this strategy offers the highest sensitivity to detect the potential effects of environmental chemicals on the nervous system. Finally, given that in our study urinary BDNF levels were associated with internalizing but not externalizing problems (the scale associated with MnBP and DEHP metabolites in boys), we did not perform mediation analyses.

### 4.5. Strengths and Limitations

Among the strengths of this study is the inclusion of five cohorts belonging to different European countries, which better reflected the differences in sociodemographic and exposure profiles in Europe. Chemical measurements were performed under the rigorous quality controls and interlaboratory comparisons within the HBM4EU project [50], promoting an optimal exposure measurement. Since urinary BDNF measurements were also previously validated and performed in the same laboratory [53], the acquisition of exposure and effect data in this study was comparable and pre-harmonized. Furthermore, this is the first study to evaluate associations between phthalate metabolites and BDNF in children, using urine as a noninvasive and readily available matrix. Among the limitations, we must highlight the cross-sectional design that prevented us from establishing the temporality of associations and is more prone to possible reverse causality issues. In addition, we did not correct for multiple comparisons since this approach may be counterproductive due to a disproportionate increase in type 2 errors [110] and because our interpretation was not based solely on statistical significance but also considered previous toxicological and epidemiological data. Although the possibility of some chance findings cannot be fully excluded, the observed associations between DnBP/DEHP metabolites and externalizing problems were in line with the weight of evidence, which increases our confidence in these results. Although data for phthalates and BDNF were available for 1148 children, behavioral data were only available for 298 children from the NACII-IT cohort. Phthalate and DINCH metabolites are short-lived compounds with rapid excretion and high variability over days/weeks. Therefore, the use of one spot urine sample may have led to exposure misclassification. However, in general, this would tend to result in attenuation bias [111,112,113,114], that is, a bias toward null findings, meaning that we would be underestimating rather than overestimating the associations with phthalate/DINCH metabolites. Finally, despite models were controlled for relevant covariates, we cannot discard possible unmeasured or residual confounders such as diet quality or physical exercise.

## 5. Conclusions

In European children aged 7–12 years, exposure to urinary concentrations of DnBP and DEHP metabolites were cross-sectionally associated with higher externalizing problems in the NACII-IT cohort, especially among boys. Higher urinary concentrations of the non-phthalate replacement DINCH were associated with lower serum BDNF levels among children of the CRP-SLO cohort. Although most urinary phthalate metabolites were associated with higher urinary BDNF protein levels when four out of the five cohorts were examined together, we identified the possibility of a “urine concentration bias” that could also explain the associations observed in noncausal terms, so these results should be carefully interpreted. Finally, higher urinary BDNF levels were associated with more internalizing problems among boys and girls from the NACII-IT cohort, suggesting that urinary BDNF levels could be used as a noninvasive biomarker to predict behavioral alterations in children. Given this is the first observational study investigating phthalate exposure in relation to BDNF biomarkers, future studies are needed to validate the associations observed.

## Figures and Tables

**Table 1:** Characteristics and data availability of included studies.

Variables	NACII-IT	PCB-SK	InAirQ-HU	NEBII-NO	CRP-SLO	Combined ^b^
	***Cohort characteristics [Mean (SD) or frequency (percentage)]***					
**Country**	Italy	Slovakia	Hungary	Norway	Slovenia	-
**Initial sample size**	300	294	257	297	124	1148
**Age**	7.0 (0.2)	11.1 (0.4)	9.3 (0.7)	9.83 (1.17)	8.85 (0.97)	9.32 (1.68)
**BMI (Kg/m^2^)**	16.9 (2.26)	20.2 (4.3)	17.4 (3.3)	17.4 (2.46)	17.2 (3.09)	18.0 (3.50)
**BMI z-score**	0.49 (1.15)	0.92 (1.22)	0.27 (1.35)	0.32 (0.81)	0.34 (1.44)	0.51 (1.17)
**Child sex = boys**	150 (50%)	130 (44%)	130 (51%)	160 (54%)	55 (44%)	570 (50%)
**Maternal education**						
Low	42 (14%)	33 (11%)	9 (4%)	0 (0%)	11 (9%)	84 (8%)
Medium	139 (46%)	235 (80%)	122 (52%)	39 (13%)	30 (24%)	535 (47%)
High	119 (40%)	25 (9%)	103 (44%)	258 (87%)	83 (67%)	505 (45%)
**Maternal IQ**	120 (10.7) [68–128]	108 (15.1) [63–132]	-	-	-	114.1 (14.5) [63–132]
	***Behavior CBCL assessment (Mean (SD)/[range] and BDNF levels (Median, P25, P75; μg/g creatinine)***					
**Total CBCL score**	23.2 (15.0) [0–87]	-	-	-	-	-
**Externalizing score**	5.4 (4.6) [0–27]	-	-	-	-	-
**Internalizing score**	6.3 (5.4) [0–34]	-	-	-	-	-
**Urinary BDNF ^a^**	2.87 (1.98, 4.21)	2.65 (1.85, 3.83)	2.52 (1.70, 3.54)	1.52 (1.08, 1.98)	-	2.31 (1.52, 3.46)
**Serum BDNF**	-	-	-	-	45.1 (38.9, 52.6)	-
	***Phthalate concentrations (Median, P25, P75; μg/g creatinine)***					
**MEP**	63.5 (33.3, 131.9)	23.9 (12.9, 54.9)	9.64 (5.82, 18.1)	14.3 (9.70, 25.6)	25.3 (15.7, 51.2)	21.8 (10.4, 54.4)
**MnBP**	21.9 (14.5, 33.0)	58.8 (35.9, 102.8)	18.6 (11.5, 30.0)	23.9 (15.1, 35.2)	19.9 (14.0, 26.9)	26.3 (15.5, 47.2)
**MiBP**	34.2 (24.6, 50.2)	48.3 (25.9, 81.0)	34.0 (19.9, 51.7)	26.7 (16.1, 41.3)	27.0 (17.5, 43.5)	33.9 (21.5, 57.7)
**MBzP**	6.69 (3.89, 11.1)	3.94 (0.77, 7.58)	1.59 (0.93, 2.84)	4.88 (2.95, 8.61)	3.47 (2.12, 5.52)	4.14 (1.80, 7.99)
**∑DEHP**	69.4 (51.8, 108.1)	88.2 (57.8, 136.6)	36.9 (23.8, 56.3)	40.8 (29.3, 62.3)	55.4 (38.4, 87.4)	56.4 (35.6, 93.4)
**MEHHP**	19.2 (14.3, 28.8)	20.3 (14.0, 31.8)	10.8 (6.85, 16.6)	9.29 (6.35, 14.5)	14.6 (9.11, 21.9)	14.8 (8.56, 23.5)
**MEOHP**	9.82 (6.89, 15.0)	18.1 (12.2, 25.6)	5.23 (3.43, 8.42)	5.74 (3.89, 8.66)	11.1 (7.27, 17.4)	8.62 (4.91, 15.4)
**MECPP**	24.2 (16.8, 37.3)	28.2 (18.0, 46.8)	11.4 (7.32, 18.3)	17.2 (12.3, 24.8)	17.7 (11.7, 25.8)	19.6 (12.5, 31.6)
**∑DINCH ^c^**	6.17 (3.40, 10.5)	2.52 (1.43, 4.97)	1.97 (1.25, 4.11)	-	4.27 (2.43, 7.05)	3.30 (1.68, 6.68)
**MHINCH**	3.65 (2.03, 6.68)	1.65 (0.90, 3.35)	1.20 (0.74, 2.42)	2.94 (1.91, 5.52)	2.61 (1.41, 4.52)	2.28 (1.23, 4.54)
**MCOCH ^c^**	2.24 (1.26, 4.13)	0.86 (0.46, 1.62)	0.90 (0.49, 1.68)	-	1.55 (0.89, 2.70)	1.21 (0.58, 2.49)

**Abbreviations:** BDNF (brain-derived neurotrophic factor); BMI (body mass index); CBCL (Child Behavior Checklist); ISCED (International Standard Classification of Education); IQ (intelligence quotient); NACII-IT (Italian Northern Adriatic Cohort II); PCB-SK (Slovakian PCB-SK cohort); InAirQ-HU (Hungarian Integrated Indoor Air Quality Management cohort); MEP (mono-ethyl phthalate); MnBP (mono-n-butyl phthalate); MiBP (mono-isobutyl phthalate); MBzP (mono-benzyl phthalate); DEHP (di(2-ethylhexyl) phthalate); MEHHP (mono(2-ethyl-5-hydroxyhexyl) phthalate); MEOHP (mono(2-ethyl-5-oxohexyl) phthalate); MECPP (mono(2-ethyl-5-carboxypentyl) phthalate); DINCH (di(isononyl)cyclohexane-1,2-dicarboxylate); MHINCH (mono-hydroxy isononyl cyclohexane-1,2-dicarboxylate); MCOCH (mono-oxo isononyl cyclohexane-1,2-dicarboxylate). ^a^ (μg/g of creatinine). ^b^ Combined data from NACII-IT, PCB-SK, InAirQ-HU and NEBII-NO cohorts, excluding CRP-SLO since the later cohort did not had data on urinary BDNF levels. ^c^ The ∑DINCH measure was estimated without considering the NEBII-NO cohort, which only had data on the MHINCH but not the MCOCH metabolite.

**Table 2:** Mixture associations using weighted quantile sum (WQS) regression.

Outcome	All	Boys	Girls
	IRR or PC (95% CI)	IRR or PC (95% CI)	IRR or PC (95% CI)
Total CBCL score	1.09 (0.96, 1.23)	1.07 (0.91, 1.25)	1.11 (0.90, 1.35)
Externalizing score	1.15 (0.97, 1.36)	1.20 (0.96, 1.49)	1.11 (0.88, 1.39)
Internalizing score	1.03 (0.90, 1.17)	1.04 (0.85, 1.29)	1.00 (0.76, 1.30)
Urinary BDNF levels	25.9 (17.6, 34.7)	36.0 (24.3, 48.9)	18.6 (7.92, 30.5)

**Note:** Sample size for CBCL was 298, 149 for boys and 149 for girls, respectively. Sample size for urinary BDNF was 1148 in total, 570 and 578 for boys and girls, respectively (since NEBII-NO participants had data on MHINCH but not MCOCH, mixture models only included the MHINCH metabolite). Mixture models between phthalate exposure and serum BDNF in the CRP-SLO cohort were not computed due to the limited sample size available. The incidence rate ratio (IRR) or percentage change (PC) represents the mean change in CBCL score or BDNF levels per tertile increase of the mixture, with its related 95% confidence interval (95% CI). The weight contributed by each chemical compound to the most relevant mixture associations can be consulted in Supplementary Figure S2. Mixture models were adjusted for the same set of covariates considered in single-pollutant analyses.

## Data Availability

Metadata of the five cohorts are available from IPCHEM, the European Commission’s Information Platform for Chemical Monitoring. The summary statistics (percentiles P5, P10, P25, P50, P75, P90, P95) of the exposure biomarkers are made available on the openly accessible online European HBM dashboard (https://hbm.vito.be/eu-hbm-dashboard, accessed on 28 August 2024) and IPCHEM (https://ipchem.jrc.ec.europa.eu/, accessed on 28 August 2024), where they can be accessed. Data sharing of individual-level data is possible upon request (https://hbm.vito.be/peh-data-platform, accessed on 28 August 2024).

## Supplementary Materials

The following supporting information can be downloaded at https://www.mdpi.com/article/10.3390/toxics12090642/s1, Figure S1: Spearman’s correlation heatplot of creatinine-standardized phthalate metabolite concentrations, Figure S2: Relative weights of phthalates for mixture associations, Figure S3: Sensitivity analysis showing adjusted cross-sectional associations between concurrent childhood urinary phthalate metabolite concentrations and urinary BDNF levels in children of the PCB-SK, InAirQ-HU and NEBII-NO cohorts, Figure S4: Sensitivity analyses comparing five different methods of creatinine adjustment, Table S1: Adjusted cross-sectional associations between concurrent childhood urinary phthalate metabolite concentrations and CBCL scores among all children in the NACII-IT cohort (n = 298), Table S2: Adjusted cross-sectional associations between concurrent childhood urinary phthalate metabolite concentrations and CBCL scores in boys and girls separately in the NACII-IT cohort (n = 298), Table S3: Adjusted cross-sectional associations between concurrent childhood urinary phthalate metabolite concentrations and urinary BDNF levels in children of the NACII-IT cohort (n = 299), Table S4: Adjusted cross-sectional associations between concurrent childhood urinary BDNF levels and CBCL scores in children of the NACII-IT cohort (n = 299), Table S5: Adjusted cross-sectional associations between concurrent childhood urinary phthalate metabolite concentrations and urinary BDNF levels in children of the NACII-IT, PCB-SK and InAirQ cohorts (N = 1148), Table S6: Adjusted cross-sectional associations between concurrent childhood urinary phthalate metabolite concentrations and serum BDNF levels in children of the CRP-SLO cohort (N = 124), Table S7: Spearman’s correlations between urinary creatinine and BDNF and phthalate/DINCH metabolite levels in NACII-IT, PCB-SK and InAirQ cohorts combined (N = 1148).
