# Supplementary material for: Associations between Urinary Phthalate Metabolites with BDNF and Behavioral Function among European Children from Five HBM4EU Aligned Studies
Source: Toxics. 2024 Aug 31;12(9):642. doi: 10.3390/toxics12090642 (PMC11436069; doi:10.3390/toxics12090642)
Supplement: Supplementary file 1 [file toxics-12-00642-s001.zip › toxics-3156928-supplementary.pdf]

Supporting Information

Associations between Urinary Phthalate Metabolites with BDNF and Behavioral Function among European Children from Five HBM4EU Aligned Studies

Elena Salamanca-Fernández, Lydia Espín-Moreno, Alicia Olivas-Martínez, Ainhoa Pérez-Cantero, José L. Martín-Rodríguez, Rafael M. Poyatos, Fabio Barbone, Valentina Rosolen, Marika Mariuz, Luca Ronfani, Ľubica Palkovičová Murínová, Lucia Fábelová, Tamás Szigeti, Réka Kakucs, Amrit K. Sakhi, Line S. Haug, Birgitte Lindeman, Janja Snoj Tratnik, Tina Kosjek, Griet Jacobs, Stefan Voorspoels, Helena Jurdáková, Renáta Górová, Ida Petrovičová, Branislav Kolena, Marta Esteban, Susana Pedraza-Díaz, Marike Kolossa-Gehring, Sylvie Remy, Eva Govarts, Greet Schoeters, Mariana F. Fernández and Vicente Mustieles

Index

Table S1, Adjusted cross-sectional associations between concurrent childhood urinary phthalate metabolite concentrations and CBCL scores among all children in the NACII-IT cohort (n=298)……………………………………………………………………………………………….....3

Table S2. Adjusted cross-sectional associations between concurrent childhood urinary phthalate metabolite concentrations and CBCL scores in boys and girls separately in the NACII-IT cohort (n=298)………………………………………………………………………………………4

Table S3. Adjusted cross-sectional associations between concurrent childhood urinary phthalate metabolite concentrations and urinary BDNF levels in children of the NACII-IT cohort (n=299)……………………………………………………………………………………………….…5

Table S4. Adjusted cross-sectional associations between concurrent childhood urinary BDNF levels and CBCL scores in children of the NACII-IT cohort (n=299)……………………6

Table S5. Adjusted cross-sectional associations between concurrent childhood urinary phthalate metabolite concentrations and urinary BDNF levels in children of the *NACII-IT. PCB-SK and InAirQ cohorts (N=1148)*………………………………………………………………...7

Table S6. Adjusted cross-sectional associations between concurrent childhood urinary phthalate metabolite concentrations and serum BDNF levels in children of the *CRP-SLO cohort (N=124)*……………………………………………………………………………………………………*8*

Table S7. Spearman correlations between urinary creatinine and BDNF and phthalate/DINCH metabolite levels in NACII-IT. PCB-SK and InAirQ cohorts combined (N=1148)………9

Figure S1. Spearman correlation heatplot of creatinine-standardized phthalate metabolite concentrations……………………………………………………………………………….10

Figure S2. Relative weights of phthalates for mixture associations…………………………11

Figure S3. Sensitivity analysis showing adjusted cross-sectional associations between concurrent childhood urinary phthalate metabolite concentrations and urinary BDNF levels in children of the PCB-SK (n=291: 127 boys and 164 girls) and InAirQ-HU (n=234: 118boys and 116 girls) cohorts……………………………………………………………………………….12

Figure S4. Set of sensitivity analyses testing different types of urinary creatinine standardization/adjustment on the cross-sectional association between urinary phthalate metabolite concentrations and urinary BDNF levels in all children of the NACII-IT, PCB-SK, InAirQ-HU and NEBII-NO cohorts combined (N=1148)………………………………………………………………13

Table S1. Adjusted cross-sectional associations between concurrent childhood urinary phthalate metabolite concentrations and CBCL scores among all children in the NACII-IT cohort (n=298).

Biomarker	All children (n=298)											
	Total score				Externalizing				Internalizing			
	IRR	95% CI	p	p-int	IRR	95% CI	p	p-int	IRR	95% CI	p	p-int
MEP	1.00	(0.93, 1.07)	0.94	0.39	0.97	(0.88, 1.07)	0.506	0.14	1.03	(0.94, 1.14)	0.513	0.30
MnBP	1.01	(0.92, 1.11)	0.891	0.27	1.01	(0.89, 1.13)	0.924	**0.01**	1.03	(0.91, 1.17)	0.652	0.76
MiBP	1.03	(0.92, 1.16)	0.574	0.69	1.05	(0.90, 1.21)	0.553	0.43	1.08	(0.93, 1.27)	0.311	0.47
MBzP	1.02	(0.93, 1.11)	0.70	0.76	1.04	(0.93, 1.16)	0.509	0.92	0.99	(0.89, 1.11)	0.925	0.36
∑DEHP	1.05	(0.93, 1.19)	0.391	0.67	1.13	(0.96, 1.32)	0.141	0.47	1.01	(0.85, 1.19)	0.935	0.77
MEHHP	1.03	(0.92, 1.16)	0.562	0.77	1.11	(0.95, 1.29)	0.186	0.53	0.97	(0.83, 1.13)	0.696	0.83
MEOHP	1.05	(0.93, 1.18)	0.416	0.49	1.14	(0.97, 1.32)	0.104	0.31	1.01	(0.86, 1.18)	0.94	0.62
MECPP	1.07	(0.95, 1.20)	0.254	0.62	1.13	(0.97, 1.31)	0.123	0.47	1.05	(0.90, 1.23)	0.542	0.51
∑DINCH	1.03	(0.95, 1.10)	0.489	0.35	1.05	(0.95, 1.15)	0.322	0.55	0.98	(0.89, 1.07)	0.602	0.19
MHINCH	1.03	(0.96, 1.10)	0.475	0.35	1.05	(0.96, 1.16)	0.282	0.47	0.97	(0.88, 1.07)	0.539	0.17
MCOCH	1.02	(0.95, 1.10)	0.525	0.38	1.04	(0.95, 1.14)	0.401	0.69	0.98	(0.90, 1.08)	0.725	0.25

Significant (p-value <0.05) and borderline (p-value <0.10) results highlighted in bold. Phthalate concentrations were natural log–transformed, so the incidence rate ratios (IRRs) and 95% Confidence Intervals (95% CI) correspond to the multiplicative change in the probability of CBCL scores for each 2.7-fold increase in exposure biomarkers. Models adjusted for: maternal education (low (0-2), middle (3-4) or high (>=5) ISCED classification), child body mass index (BMI) z-score, child sex, creatinine (log-transformed), child age at CBCL assessment, season of urine collection (summer/autumn vs. winter/spring) and maternal IQ.

Table S2. Adjusted cross-sectional associations between concurrent childhood urinary phthalate metabolite concentrations and CBCL scores in boys and girls separately in the NACII-IT cohort (n=298).

Biomarker	Boys (n=149)									Girls (n=149)								
	Total score			Externalizing			Internalizing			Total score			Externalizing			Internalizing		
	IRR	95% CI	p	IRR	95% CI	p	IRR	95% CI	p	IRR	95% CI	p	IRR	95% CI	p	IRR	95% CI	p
MEP	1.02	(0.93, 1.13)	0.62	1.04	(0.92, 1.17)	0.53	1.07	(0.93, 1.22)	0.35	0.97	(0.87, 1.09)	0.60	0.90	(0.77, 1.05)	0.17	1.01	(0.87, 1.16)	0.94
MnBP	1.07	(0.94, 1.21)	0.30	**1.20**	**(1.02, 1.42)**	**0.03**	1.05	(0.89, 1.24)	0.56	0.96	(0.83, 1.10)	0.55	0.89	(0.74, 1.08)	0.23	1.00	(0.81, 1.22)	0.98
MiBP	1.01	(0.87, 1.19)	0.87	0.96	(0.78, 1.19)	0.73	1.14	(0.92, 1.41)	0.23	1.07	(0.90, 1.26)	0.46	1.13	(0.91, 1.41)	0.28	1.00	(0.79, 1.27)	0.98
MBzP	1.03	(0.92, 1.15)	0.62	1.03	(0.90, 1.20)	0.64	1.03	(0.89, 1.20)	0.68	1.01	(0.88, 1.15)	0.94	1.05	(0.88, 1.26)	0.60	0.93	(0.78, 1.11)	0.41
∑DEHP	1.10	(0.93, 1.31)	0.26	**1.23**	**(1.00, 1.53)**	**0.05**	1.05	(0.82, 1.34)	0.72	1.03	(0.87, 1.23)	0.72	1.07	(0.85, 1.36)	0.56	0.97	(0.77, 1.22)	0.79
MEHHP	1.07	(0.91, 1.26)	0.42	**1.20**	**(0.98, 1.48)**	**0.08**	0.96	(0.76, 1.21)	0.73	1.02	(0.87, 1.20)	0.82	1.06	(0.85, 1.32)	0.61	0.97	(0.78, 1.20)	0.77
MEOHP	1.11	(0.95, 1.31)	0.20	**1.26**	**(1.03, 1.55)**	**0.03**	1.06	(0.84, 1.34)	0.60	1.01	(0.85, 1.20)	0.92	1.05	(0.83, 1.32)	0.70	0.96	(0.77, 1.19)	0.69
MECPP	1.13	(0.96, 1.32)	0.16	**1.23**	**(1.00, 1.51)**	**0.05**	1.13	(0.89, 1.44)	0.30	1.05	(0.89, 1.24)	0.58	1.08	(0.86, 1.36)	0.49	0.99	(0.80, 1.22)	0.91
∑DINCH	0.98	(0.88, 1.10)	0.76	1.00	(0.87, 1.16)	0.95	0.91	(0.78, 1.06)	0.21	1.05	(0.95, 1.16)	0.33	1.08	(0.95, 1.23)	0.26	1.01	(0.89, 1.14)	0.88
MHINCH	0.98	(0.88, 1.10)	0.75	1.00	(0.87, 1.16)	0.97	0.9	(0.77, 1.04)	0.16	1.05	(0.95, 1.16)	0.32	1.08	(0.95, 1.23)	0.21	1.01	(0.89, 1.14)	0.90
MCOCH	0.99	(0.89, 1.10)	0.80	1.01	(0.88, 1.15)	0.91	0.93	(0.81, 1.08)	0.35	1.05	(0.95, 1.15)	0.36	1.06	(0.93, 1.21)	0.37	1.01	(0.89, 1.15)	0.84

Significant (p-value <0.05) and borderline (p-value <0.10) results highlighted in bold. Phthalate concentrations were natural log–transformed, so the incidence rate ratios (IRRs) and 95% Confidence Intervals (95% CI) correspond to the multiplicative change in the probability of CBCL scores for each 2.7-fold increase in exposure biomarkers. Models adjusted for: maternal education (low (0-2), middle (3-4) or high (>=5) ISCED classification), child body mass index (BMI) z-score, creatinine (log-transformed), child age at CBCL assessment, season of urine collection (summer/autumn vs. winter/spring) and maternal IQ.

Table S3. Adjusted cross-sectional associations between concurrent childhood urinary phthalate metabolite concentrations and urinary BDNF levels in children of the NACII-IT cohort (n=299).

**Biomarker**	**All children (n=299)**				**Boys (n=150)**			**Girls (n=149)**		
	**Urinary BDNF**				**Urinary BDNF**			**Urinary BDNF**		
	**PC**	**95% CI**	**p**	**p-int**	**PC**	**95% CI**	**p**	**PC**	**95% CI**	**p**
MEP	**8.43**	**(-0.18, 17.78)**	**0.05**	0.32	**13.62**	**(3.23, 25.07)**	**0.01**	1.67	(-11.42, 16.70)	0.81
MnBP	**21.19**	**(8.74, 35.07)**	**0.00**	0.51	**22.46**	**(7.92, 38.97)**	**0.00**	**17.68**	**(-2.37, 41.86)**	**0.09**
MiBP	**27.82**	**(11.77, 46.17)**	**0.00**	0.20	**40.88**	**(21.79, 62.96)**	**0.00**	12.06	(-11.42, 41.78)	0.34
MBzP	**14.4**	**(3.93, 25.92)**	**0.01**	0.75	**17.62**	**(5.55, 31.08)**	**0.00**	10.39	(-6.72, 30.64)	0.25
∑DEHP	7.99	(-5.76, 23.74)	0.27	0.34	10.38	(-6.76, 30.66)	0.25	4.38	(-15.91, 29.56)	0.70
MEHHP	1.97	(-10.41, 16.07)	0.77	0.43	1.57	(-13.80, 19.68)	0.85	1.21	(-17.26, 23.81)	0.91
MEOHP	**14.01**	**(0.11, 29.85)**	**0.05**	0.46	**19.18**	(1.88, 39.42)	**0.03**	8.84	(-11.72, 34.18)	0.43
MECPP	12.79	(-0.83, 28.28)	0.07	0.43	15.8	(-1.24, 35.77)	0.07	8.19	(-11.88, 32.82)	0.45
∑DINCH	-5.02	(-12.40, 2.99)	0.21	0.38	-0.49	(-10.76, 10.97)	0.93	-8.68	(-18.99, 2.93)	0.14
MHINCH	-4.88	(-12.23, 3.09)	0.22	0.42	-0.69	(-10.88, 10.68)	0.90	-8.24	(-18.57, 3.39)	0.16
MCOCH	-5.05	(-12.30, 2.79)	0.20	0.36	-0.42	(-10.48, 10.77)	0.94	-8.89	(-18.98, 2.46)	0.12

Significant (p-value <0.05) and borderline (p-value <0.10) results highlighted in bold. Phthalate concentrations were natural log–transformed, so the percent change (PC) and 95% Confidence Intervals (95% CI) correspond to the mean change in the urinary BDNF levels for each 2.7-fold increase in exposure biomarkers. Models adjusted for: maternal education (low (0-2), middle (3-4) or high (>=5) ISCED classification), child body mass index (BMI) z-score, creatinine (log-transformed), child sex, child age at urine collection and season of urine collection (summer/autumn vs. winter/spring).

Table S4. Adjusted cross-sectional associations between concurrent childhood urinary BDNF levels and CBCL scores in children of the NACII-IT cohort (n=299).

Biomarker	Total score			Externalizing			Internalizing		
	IRR	95% CI	p	IRR	95% CI	p	IRR	95% CI	p
BDNF_all children	1.03	(0.93, 1.15)	0.54	0.98	(0.85, 1.12)	0.78	**1.15**	**(1.00, 1.32)**	**0.05**
BDNF_boys (n=150)	0.99	(0.82, 1.18)	0.87	0.92	(0.73, 1.15)	0.44	1.12	(0.87, 1.43)	0.38
BDNF_girls (n=149)	1.06	(0.93, 1.21)	0.41	1.03	(0.86, 1.23)	0.74	**1.16**	**(0.97, 1.38)**	**0.10**

Significant (p-value <0.05) and borderline (p-value <0.10) results highlighted in bold. BDNF levels were natural log–transformed, so the incidence rate ratios (IRRs) and 95% Confidence Intervals (95% CI) correspond to the multiplicative change in the probability of CBCL scores for each 2.7-fold increase in urinary BDNF levels. Models adjusted for: maternal education (low (0-2), middle (3-4) or high (>=5) ISCED classification), child body mass index (BMI) z-score, creatinine (log-transformed), child age at CBCL assessment, season of urine collection (summer/autumn vs. winter/spring) and maternal IQ.

Table S5. Adjusted cross-sectional associations between concurrent childhood urinary phthalate metabolite concentrations and urinary BDNF levels in children of the *NACII-IT. PCB-SK. InAirQ and NEBII-NO cohorts (N=1148).*

Biomarker	All children			Boys (n=570)			Girls (n=578)		
	Urinary BDNF			Urinary BDNF			Urinary BDNF		
	**PC**	**95% CI**	**p**	**PC**	**95% CI**	**p**	**PC**	**95% CI**	**p**
MEP	**4.15**	**(0.20, 8.25)**	**0.04**	**6.86**	**(1.77, 12.19)**	**0.01**	0.81	(-5.05, 7.03)	0.79
MnBP	**18.99**	**(12.71, 25.63)**	**0.00**	**20.83**	**(12.90, 29.33)**	**0.00**	**15.54**	**(6.11, 25.80)**	**0.00**
MiBP	**14.4**	**(8.64, 20.48)**	**0.00**	**22.48**	**(15.03, 30.41)**	**0.00**	4.29	(-4.13, 13.46)	0.33
MBzP	-1.88	(-5.43, 1.81)	0.31	-3.14	(-7.52, 1.44)	0.18	-1.37	(-6.93, 4.53)	0.64
∑DEHP	**12.95**	**(6.83, 19.42)**	**0.00**	**17.89**	**(10.34, 25.97)**	**0.00**	5.7	(-3.64, 15.94)	0.24
MEHHP	**13.53**	**(7.67, 19.70)**	**0.00**	**17.62**	**(10.44, 25.27)**	**0.00**	7.35	(-1.68, 17.22)	0.11
MEOHP	**17.54**	**(11.56, 23.83)**	**0.00**	**22.1**	**(14.84, 29.82)**	**0.00**	**10.73**	**(1.45, 20.86)**	**0.02**
MECPP	**9.42**	**(3.50, 15.67)**	**0.00**	**14.29**	**(6.88, 22.22)**	**0.00**	2.56	(-6.36, 12.33)	0.58
∑DINCH	-3.10	(-7.10, 1.06)	0.14	1.18	(-3.81, 6.42)	0.65	**-8.44**	**(-14.30, -2.17)**	**0.01**
MHINCH	**-6.69**	**(-10.38, -2.85)**	**0.00**	-2.59	(-7.50, 2.59)	0.32	**-11.26**	**(-16.60, -5.57)**	**0.00**
MCOCH	**-3.55**	**(-7.47, 0.53)**	**0.09**	0.27	(-4.55, 5.33)	0.92	**-8.43**	**(-14.25, -2.22)**	**0.01**

Significant (p-value <0.05) and borderline (p-value <0.10) results highlighted in bold. Phthalate concentrations were natural log–transformed, so the percent change (PC) and 95% Confidence Intervals (95% CI) correspond to the mean change in the urinary BDNF levels for each 2.7-fold increase in exposure biomarkers. Models adjusted for: maternal education (low (0-2), middle (3-4) or high (>=5) ISCED classification), child body mass index (BMI) z-score, creatinine (log-transformed), child sex, child age at urine collection and season of urine collection (summer/autumn vs. winter/spring).

Table S6. Adjusted cross-sectional associations between concurrent childhood urinary phthalate metabolite concentrations and serum BDNF levels in children of CRP-SLO *cohort (N=124).*

Biomarker	All children (N=124)			Boys (n=55)			Girls (n=69)		
	Serum BDNF			Serum BDNF			Serum BDNF		
	PC	95% CI	p	PC	95% CI	p	PC	95% CI	p
MEP	-0.43	(-4.20, 3.49)	0.83	-1.23	(-7.76, 5.76)	0.72	0.01	(-5.09, 5.39)	1.00
MnBP	-3.11	(-10.07, 4.39)	0.40	-8.66	(-19.38, 3.48)	0.15	1.56	(-8.40, 12.60)	0.77
MiBP	**-4.70**	**(-9.81, 0.69)**	**0.09**	-2.47	(-10.77, 6.60)	0.57	**-6.83**	**(-14.04, 0.98)**	**0.08**
MBzP	-1.21	(-5.86, 3.68)	0.62	-1.02	(-8.86, 7.49)	0.80	-0.79	(-7.18, 6.04)	0.81
∑DEHP	-2.56	(-9.08, 4.43)	0.46	-8.36	(-19.29, 4.05)	0.17	0.86	(-7.66, 10.15)	0.85
MEHHP	-2.16	(-8.24, 4.33)	0.50	-7.48	(-17.75, 4.06)	0.19	0.92	(-6.99, 9.51)	0.82
MEOHP	-2.30	(-8.55, 4.38)	0.49	-7.55	(-17.99, 4.22)	0.19	0.96	(-7.22, 9.86)	0.82
MECPP	-2.01	(-8.50, 4.93)	0.56	-7.76	(-19.05, 5.11)	0.22	1.19	(-7.07, 10.18)	0.78
∑DINCH	-1.12	(-5.87, 3.88)	0.65	**-8.84**	**(-16.67, -0.27)**	**0.04**	2.44	(-3.84, 9.13)	0.45
MHINCH	-0.75	(-5.38, 4.10)	0.75	**-7.93**	**(-15.60, 0.44)**	**0.06**	2.62	(-3.49, 9.12)	0.40
MCOCH	-1.87	(-6.68, 3.19)	0.46	**-10.1**	**(-17.98, -1.54)**	**0.02**	1.83	(-4.52, 8.61)	0.57

Significant (p-value <0.05) and borderline (p-value <0.10) results highlighted in bold. Phthalate concentrations were natural log–transformed, so the percent change (PC) and 95% Confidence Intervals (95% CI) correspond to the mean change in serum BDNF levels for each 2.7-fold increase in exposure biomarkers. Models adjusted for: maternal education (low (0-2), middle (3-4) or high (>=5) ISCED classification), child body mass index (BMI) z-score, creatinine (log-transformed), child sex, child age at urine collection and season of urine collection (summer/autumn vs. winter/spring).

Table S7. Spearman correlations between urinary creatinine and BDNF and phthalate/DINCH metabolite levels in NACII-IT, PCB-SK, InAirQ-HU and NEBII-NO cohorts combined.

Variables	Creatinine
**Creatinine**	1.000
**BDNF**	0.258***
**MEP**	0.385***
**MnBP**	0.582***
**MiBP**	0.586***
**MBzP**	0.373***
**MEHHP**	0.550***
**MEOHP**	0.564***
**MECPP**	0.561***
**MHINCH**	0.285***
**MCOCH**	0.259***

*** $p < 0.001$

Figure S1. Spearman correlation heatplot of creatinine-standardized phthalate metabolite concentrations NACII-IT, PCB-SK, InAirQ-HU and NEBII-NO cohorts combined.

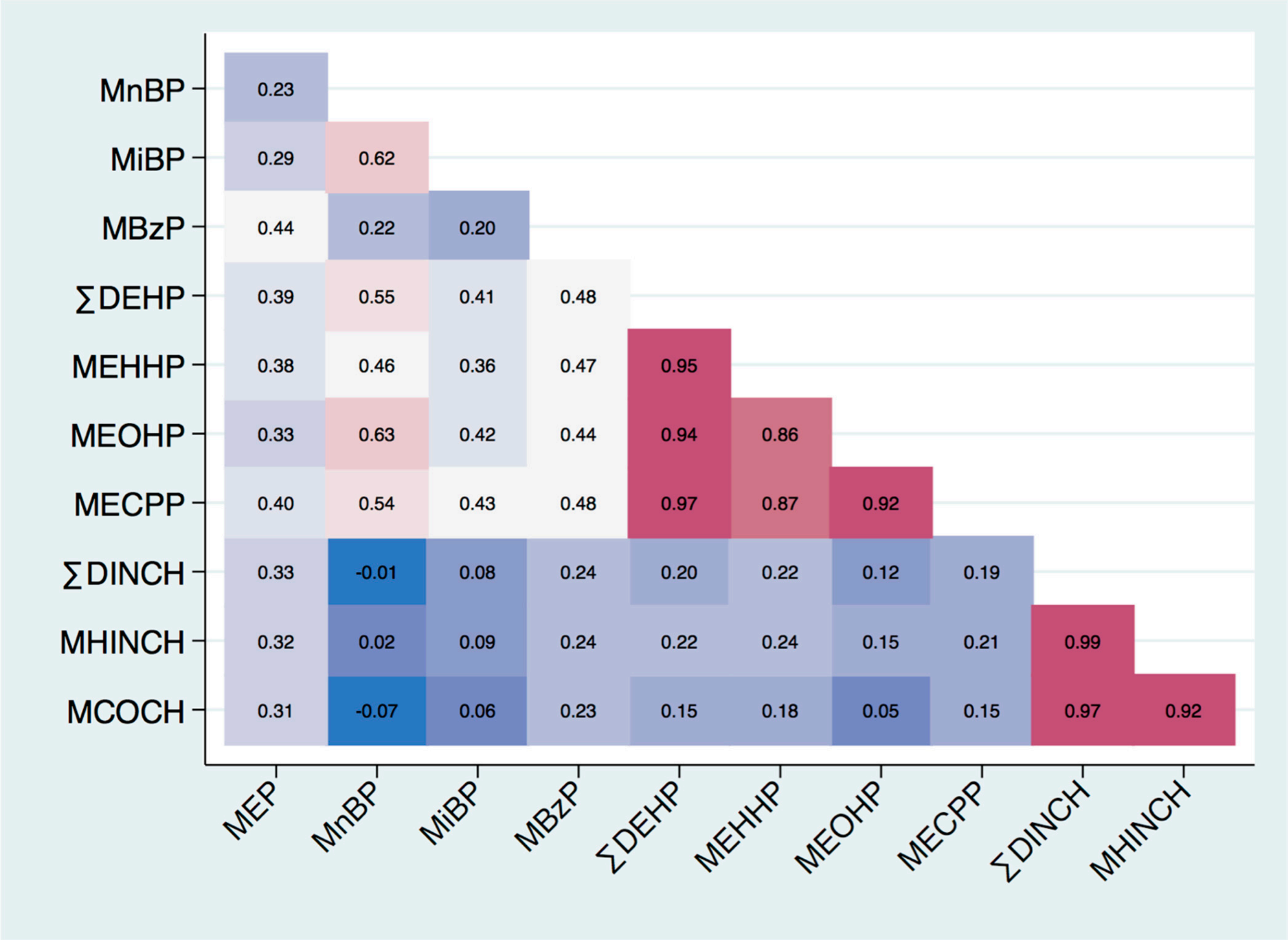

Figure S2. Relative weights of phthalates for mixture associations.

Externalizing problems in all NACII-IT children (n=298)

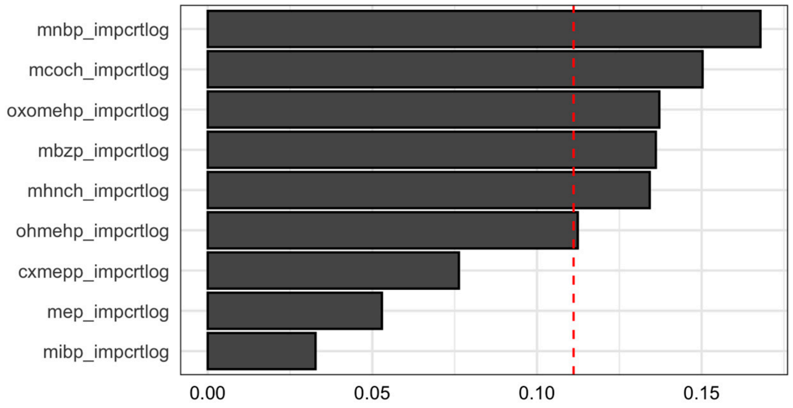

Externalizing problems in NACII-IT boys (n=149)

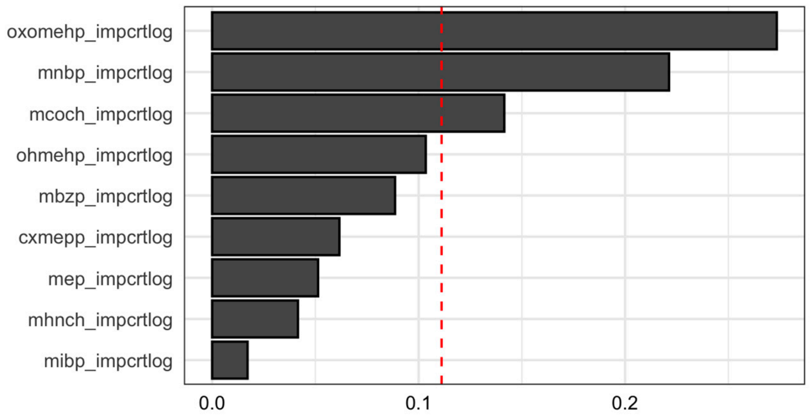

Urinary BDNF levels in all children (N=1148)

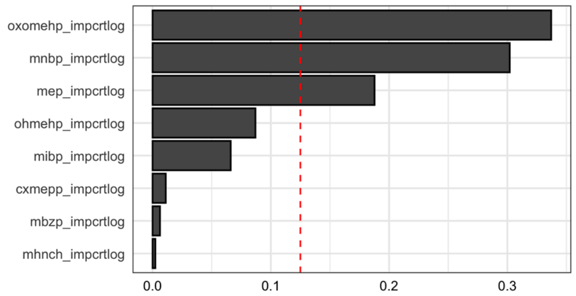

Urinary BDNF levels in boys (N=570)

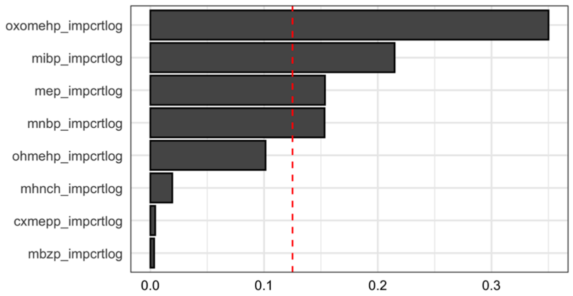

Urinary BDNF levels in girls (N=578)

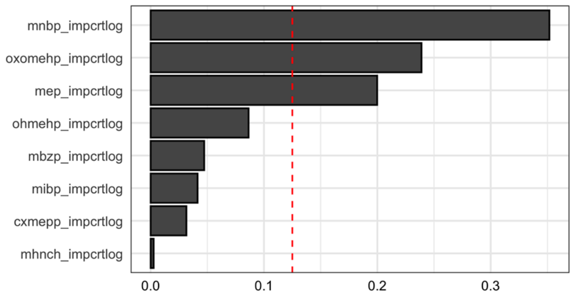

Note: Models on urinary BDNF levels included four out of the five cohorts examined including NACII-IT, PCB-SK, InAirQ-HU and NEBII-NO cohorts. Since the NEBII-NO cohort had data for the DINCH metbaolite MHINCH but not on MCOCH, mixture models only included MHINCH to avoid the exclusion of NEBII-NO from this specific analysis. Mixture models were adjusted for the same set of covariates than in single-pollutant models.

Figure S3. Sensitivity analysis showing adjusted cross-sectional associations between concurrent childhood urinary phthalate metabolite concentrations and urinary BDNF levels in children of the PCB-SK (n=291: 127 boys and 164 girls). InAirQ-HU (n=234: 118boys and 116 girls) and NEBII-NO (n=297: 160 boys and 137 girls) cohorts.

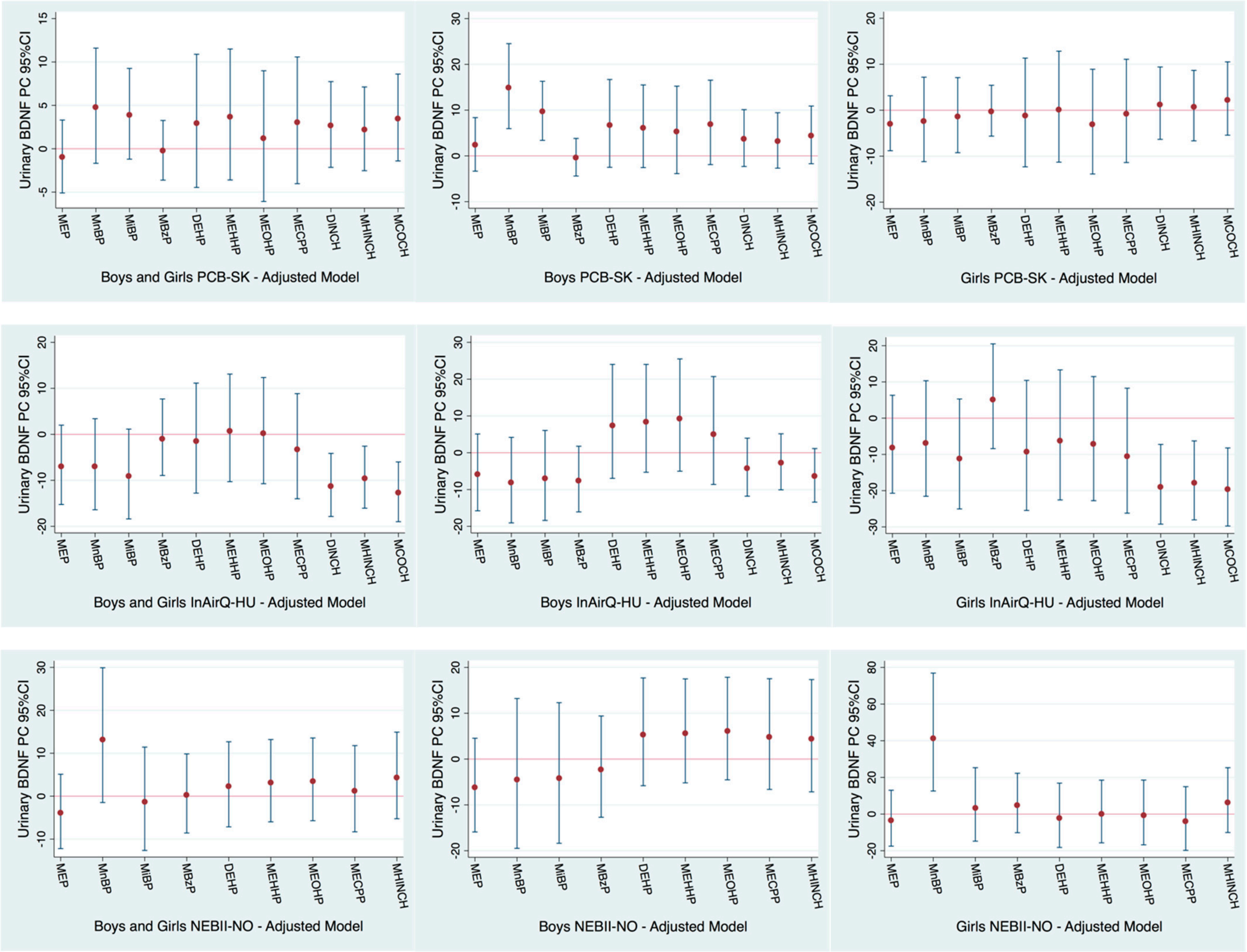

Phthalate concentrations were natural log–transformed, so the percent change (PC) and 95% Confidence Intervals (95% CI) correspond to the mean change in the urinary BDNF levels for each 2.7-fold increase in exposure biomarkers. Models adjusted for: maternal education (low (0-2), middle (3-4) or high (>=5) ISCED classification), child body mass index z-score (BMI z-score), creatinine (log-transformed), child sex, child age at urine collection and season of urine collection (summer/autumn vs. winter/spring). **Note:** Table S3 and Figure 2 show adjusted associations between phthalate metabolites and BDNF levels in children of the NACII-IT cohort.

Figure S4. Set of sensitivity analyses testing different types of urinary creatinine standardization/adjustment on the cross-sectional association between urinary phthalate metabolite concentrations and urinary BDNF levels in all children of the NACII-IT, PCB-SK, InAirQ-HU and NEBII-NO cohorts combined (N=1148).

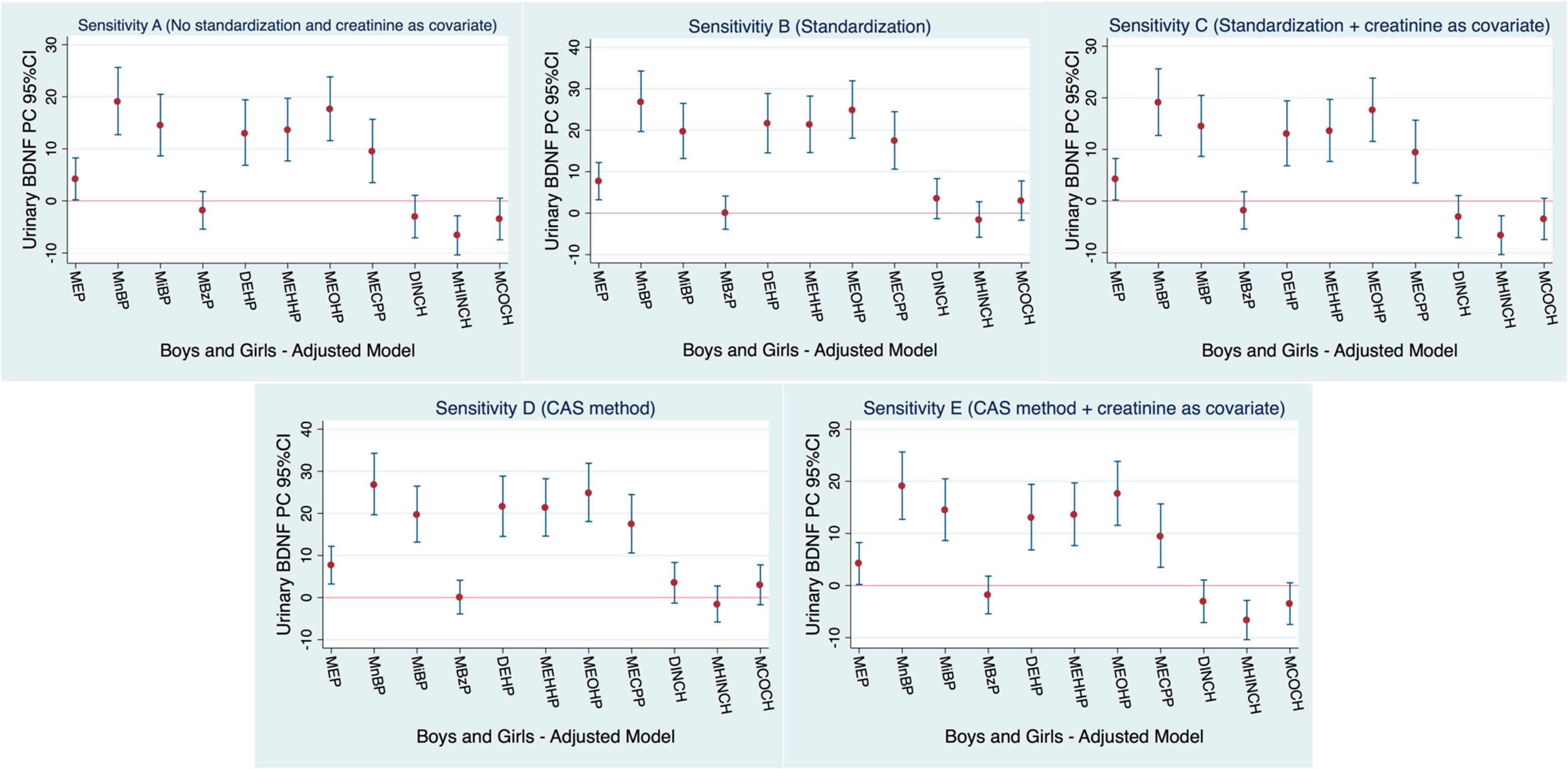

Models adjusted for: maternal education, child body mass index z-score (BMI z-score), child sex, child age at urine collection and season of urine collection (summer/autumn vs. winter/spring).

Sensitivity A: Log-transformed raw urinary phthalate metabolites and BDNF concentrations with creatinine adjustment in the model.

Sensitivity B: Log-transformed creatinine-standardized urinary phthalate metabolites and BDNF concentrations with no creatinine adjustment in the model.

Sensitivity C (Main Model): Log-transformed creatinine-standardized urinary phthalate metabolites and BDNF concentrations with creatinine adjustment in the model.

Sensitivity D: Log-transformed urinary phthalate metabolites and BDNF concentrations according to the covariate-adjusted standardization (CAS) method (O'Brien et al. 2016) with no creatinine adjustment in the model.

Sensitivity E: Log-transformed urinary phthalate metabolites and BDNF concentrations according to the covariate-adjusted standardization (CAS) method (O'Brien et al. 2016) with creatinine adjustment in the model.

Note: CAS method calculated considering age, sex and BMI z-score as causal predictors of log-transformed creatinine according to the formula presented by O'Brien et al. 2016.

O'Brien KM, Upson K, Cook NR, Weinberg CR. Environmental Chemicals in Urine and Blood: Improving Methods for Creatinine and Lipid Adjustment. Environ Health Perspect. 2016 Feb;124(2):220-7. doi: 10.1289/ehp.1509693.
